# Modulation of Growth Performance and Intestinal Microbiota in Chickens Fed Plant Extracts or Virginiamycin

**DOI:** 10.3389/fmicb.2019.01333

**Published:** 2019-06-18

**Authors:** Nianhua Zhu, Jun Wang, Longfei Yu, Qiman Zhang, Kai Chen, Baosheng Liu

**Affiliations:** ^1^College of Animal Science and Technology, Jiangxi Agricultural University, Nanchang, China; ^2^Guangdong Ruisheng Technology Co., Ltd., Guangzhou, China

**Keywords:** plant extracts, virginiamycin, intestinal microbiota, growth performance, broilers

## Abstract

In this study, the effects of plant extracts (PEs) and virginiamycin (VIRG) on broiler growth performance, as well as on host intestinal microbiota composition and function were investigated. A total of 288 one-day-old male Cobb broiler chickens were randomly divided into four treatment groups (with six replicates per group). The duodenal, ileal, and cecal content of six broilers per treatment group after 14 and 28 days of treatment were sampled. This material was used for high-throughput Illumina sequencing of the V3–V4 region of the 16S rRNA gene. The results showed that chickens fed 400 mg/kg plant extracts (HPE group) had significantly higher average body weights at day 28 as compared to the control group (CT; *P* < 0.05), and lower feed-to-meat ratios over days 15–42 (*P* < 0.01). Within the HPE group at day 14, the relative abundances of two bacterial phyla and 10 bacterial genera increased significantly in the ileal microbiota, and the relative abundance of three bacterial phyla and four bacterial genera decreased. The relative abundance of the genus *Lactobacillus* in the cecal microbiota decreased from 21.48% (CT group) to 8.41% (fed 200 mg/kg PEs; LPE group), 4.2% (HPE group), and 6.58% (fed 30 mg/kg virginiamycin; VIRG group) after 28 days. In contrast, *Faecalibacterium* and unclassified Rikenellaceae increased in abundance in the HPE group (from 18 to 28.46% and from 10.83 to 27.63%, respectively), while *Bacteroides* (36.7%) and *Lachnospiraceae* increased in abundance in the VIRG group. PICRUSt function analysis showed that the ileal microbiota of the PE treatment groups were more enriched in genes related to the meolism of cofactors and vitamins. In addition, the cecal microbiotas of the LPE and HPE groups were enriched in genes predicted to encode enzymes within 15 and 20 pathways, respectively. These pathways included protein digestion and absorption, amino acid metabolism, lipid biosynthesis, lipopolysaccharide biosynthesis, the citrate cycle (TCA cycle), and lipoic acid metabolism. Similarly, the VIRG group was enriched in 55 metabolic pathways (17 in the duodenum, 18 in the ileum, and 20 in the cecum) on day 28 (*P* < 0.05). Thus, the results indicated that the observed increase in broiler growth performance after PE or VIRG supplementation might be attributed to an improvement in intestinal microbial composition and metabolic function.

## Introduction

Subtherapeutic levels of antibiotics have been widely used in the swine and poultry industries to improve weight gain and feed conversion efficiency (Cromwell, 2002; Dibner and Richards, 2005). However, there has been increasing concern about the health and environmental safety risks of this practice, particularly due to the persistence of antibiotic residues and the transmission of antibiotic resistance. For these reasons, the use of antibiotics as feed additives has been restricted or even banned in many countries (Diarra and Malouin, 2014). Recent studies have therefore attempted to develop alternative approaches with which to maintain production performance and reduce mortality in the poultry industry (Salaheen et al., 2017). Among the available alternatives, plant extracts (PEs) or phytogenic additives have been identified as appropriate candidates due to their safety and effective antibacterial behavior (Diaz Carrasco et al., 2016; Ran et al., 2016).

Plant extracts, are essential oils that are extracted from plant materials (e.g., flowers, leaves, roots, and fruits) via solvent extraction or steam distillation. PEs are primarily composed of terpenes, terpene derivatives, and mixtures of various other compounds (Baser and Demirci, 2007; Brenes and Roura, 2010; Li et al., 2018). Due to the complexity of the PE compounds, the extracts have diverse biological effects (Diaz Carrasco et al., 2016). For example, some PE treatments have been shown to increase body weight gain and decrease the feed-to-gain ratio of broilers (Jamroz and Kamel, 2002; Ciftici et al., 2005; Jang et al., 2007; Windisch et al., 2008; Zhang et al., 2009; Khattak et al., 2014; Zeng et al., 2015). Although the specific mechanisms by which PEs improve animal growth performance have yet to be elucidated, it is clear that some PEs reduce pathogenic stress in the gut, increase digestive enzyme secretions, improve nutrient absorption, act as antioxidants, and support host immunity; these properties help to explain beneficial effects of PEs observed in the livestock industries (Windisch et al., 2008; Awaad et al., 2014; Wlodarska et al., 2015; Zeng et al., 2015). The well-known antibacterial properties of PEs have been widely tested against a wide range of pathogenic bacteria using cultures, denaturing gradient gel electrophoresis (DGGE), or PCR (Brenes and Roura, 2010; Yin et al., 2017). Recently, Li et al. (2018) used high-throughput 16S rRNA sequencing methods to investigate the effects of dietary supplementation with carvacrol and thymol on the microbial composition and metabolites in colons of weaned piglets. However, studies of the effects of PEs on the growth performance and the correlation between it and the change of intestinal microbiotas in chickens are limited.

Although the underlying mechanisms by which antibiotics enhance animal performance remain unclear, the effects of antibiotics on the host gut microbiota have been established (Pourabedin et al., 2015). Importantly, it was shown that antibiotics improved animal growth by regulating the intestinal microbiota (Diarra and Malouin, 2014). Therefore, in the present study, it was hypothesized that PEs and/or VIRG would improve broiler growth performance by modulating the composition and function of the host intestinal microbiota. This study aimed to investigate the effects of PEs and VIRG on broiler growth performance and intestinal microbiota, using high-throughput Illumina sequencing of the 16S rRNA gene.

## Materials and Methods

### PE Chemical Composition

A commercial PE product was used, which contained 20% carvacrol and 25% thymol as active components, 35% silicon dioxide as a caking inhibitor, and 12% glycerides as stabilizing agents (Guangdong Ruisheng Technology Co., Ltd., Guangdong, China).

### Experimental Animals, Diets, and Treatments

This study was approved by the Animal Care and Use Committee of China Agricultural University, permit number SYXK20171208. A total of 288 one-day-old male Cobb 500 broiler chickens were randomly divided into four groups, with six replicates per group (*n* = 12 chickens per replicate). Each group was fed one of the following four diets: CT, the basal diet, without any added growth promoter; VIRG, the basal diet supplemented with virginiamycin (30 mg/kg); LPE, the basal diet supplemented with 200 mg/kg PEs; or HPE, the basal diet supplemented with 400 mg/kg PEs. The components and nutritional composition of the basal diets are given in Table 1. The basal diets met the nutrient requirements of the National Research Council [NRC] (1994).

**Table 1:** Dietary compositions and nutrient levels of broilers (as-fed basis).

Ingredient (%)	Starter	Grower	Finisher
	(1–14 days)	(15–28 days)	(29–42 days)
Corn	55.00	57.65	59.03
Soybean meal	38.00	34.87	33.80
Soybean oil	3.00	3.50	4.00
Dicalcium phosphate	1.85	1.60	1.70
Limestone	1.17	1.20	1.30
Salt	0.35	0.35	0.35
Vitamin premix^1^	0.03	0.03	0.03
Choline chloride (50%)	0.10	0.07	0.07
Mineral premix^2^	0.20	0.20	0.18
DL-Met	0.25	0.16	0.09
HCl-Lys	0.05	0.04	0.03
Calculated nutrition levels, %			
ME, MJ/Kg	12.16	12.78	12.82
CP	20.74	19.60	18.00
Met + Cys	0.92	1.05	0.64
Lys	1.1	0.95	0.9
Ca	1.08	0.95	0.9
P	0.71	0.64	0.55

*^*1*^Vitamin premix containing the following per kilogram of diet: vitamin A, 10,000 IU; vitamin D_*3*_ (cholecalciferol), 3,500 IU; vitamin E (DL-α-tocopheryl acetate), 60 mg; vitamin K (menadione), 3 mg; thiamine, 3 mg; riboflavin, 6 mg; pyridoxine, 5 mg; vitamin B_*12*_ (cyanocobalamin), 0.01 mg; niacin, 45 mg; pantothenic acid (D-calcium pantothenate), 11 mg; folic acid, 1 mg; biotin, 0.15 mg; choline chloride, 500 mg; ethoxyquin (antioxidant), 150 mg. ^*2*^Mineral premix containing the following per kilogram of diet: Fe, 60 mg; Mn, 100 mg; Zn, 60 mg; Cu, 10 mg; I, 1 mg; Co, 0.2 mg; Se, 0.15 mg.*

### Chicken Management and Growth Performance

Chickens were reared in battery pens (1.0 × 0.8 × 0.4 m) with plastic wire floors and two water nipples per pen. Chickens had access to food and water *ad libitum.* The room temperature was maintained at 32°C for the first week, reduced evenly over the next week to 24°C, and maintained at 24°C from day 14 until the end of the experiment. The daily photoperiod was 20 h light: 4 h dark, and chickens were conventionally vaccinated against the Newcastle disease virus on day 7. Feed intake and weight gain for each replicate were measured on days 14, 28, and 42. The feed conversion ratio (FCR) was calculated for days 0–14, 15–28, and 29–42.

### Sample Collection

On days 14 and 28, six chickens per treatment group (one bird per replicate) were randomly selected, and each selected bird was intravenously injected with sodium pentobarbital (30 mg/kg body weight). Chickens were killed using jugular exsanguinations, and 2 g of digesta from the duodenum, ileum, and cecum of each bird was taken. The digesta samples were mixed, collected in sterilized tubes, immediately frozen in liquid nitrogen, and stored at -80°C.

### DNA Extraction

Total bacterial genomic DNA was extracted from each digesta sample using a FastDNA SPIN extraction kit (MP Biomedicals, Santa Ana, CA, United States), following the manufacturer’s instructions. DNA quantity and quality were determined using a NanoDrop ND-1000 spectrophotometer (Thermo Fisher Scientific, Waltham, MA, United States) and agarose gel electrophoresis respectively. DNA samples were stored at -20°C.

### 16S rDNA Amplicon Pyrosequencing

The V3–V4 region of the bacterial 16S rRNA genes was PCR amplified using the universal primers 338F (5′-ACTCCTAC GGGAGGCAGCA-3′) and 806R (5′-GGACTACHVGGGTWT CTAAT-3′), following She et al. (2018). Briefly, sample-specific 7-bp barcodes were incorporated into the primers for multiplex sequencing. Each PCR was performed in a 25-μl volume containing 2 μL DNA template, forward and reverse primers, dNTPs, DNA Polymerase, reaction buffer, and ddH_2_O. The cycling conditions were an initial denaturation at 98°C for 2 min; 25 cycles of 15 s at 98°C, 30 s at 55°C, and 30 s at 72°C; and a final extension of 5 min at 72°C. PCR amplicons were purified with Agencourt AMPure Beads (Beckman Coulter, Indianapolis, IN, United States), and quantified using the PicoGreen dsDNA Assay Kit (Invitrogen, Carlsbad, CA, United States). Purified amplicons were pooled in equal amounts, and then paired-end sequenced on an Illlumina MiSeq platform using the MiSeq Reagent Kit v3 at Shanghai Personal Biotechnology Co., Ltd. (Shanghai, China).

### Sequence Processing and Analysis

The sequencing data were processed using the Quantitative Insights Into Microbial Ecology (QIIME, v1.8.0) pipeline, following Caporaso et al. (2010). Briefly, raw sequencing reads with complete barcode matches were assigned to the appropriate sample and identified as valid, Sequences < 150 bp long, with average Phred scores < 20, containing ambiguous bases, or with mononucleotide repeats longer than 8 bp were considered low-quality and excluded from further analysis (Gill et al., 2006; Chen and Jiang, 2014). Paired-end reads were assembled using FLASH (Magoč and Salzberg, 2011). After chimera detection with QIIME, UCLUST (Edgar, 2010) was used to group the remaining high-quality sequences into operational taxonomic units (OTUs) based on a minimum sequence identity of 97%. A representative sequence was identified for each OTU using default parameters. The OTUs were taxonomically classified using BLAST; each representative sequence was searched the against the Greengenes Database (DeSantis et al., 2006) and the best hit was selected (Altschul et al., 1997). An OTU table was used to record the abundance and taxonomic affiliation of each OTU in each sample. OTUs representing less than 0.001% of all of the sequences across all of the samples were ignored. To maintain a constant sequencing depth across all of the samples, an averaged, rounded, rarefied OTU table was generated by re-sampling an average of 100 OTU subsets at a minimum sequencing depth of 90%. This averaged OTU table was used for all of the subsequent analyses.

### Bioinformatics Analysis

Bioinformatics analyses of the sequence data were primarily performed in QIIME and R. v3.2.0. OTU-level alpha diversity indices, such as the Chao1 richness estimator, Shannon diversity index, Simpson index, and Abundance-based Coverage Estimator (ACE), were calculated based on the OTU table in QIIME, and OTU-level rank abundance curves were generated to compare the evenness and richness of OTUs among samples. The numbers of OTUs unique and shared among samples or groups, irrespective of abundance, were visualized using the VennDiagram R package (Zaura et al., 2009). The relative abundances of the recovered phyla, classes, orders, families, genera and species were statistically compared among samples and groups using Metastats (White et al., 2009). Linear discriminant analysis effect size (LEfSe) analysis was performed with default parameters in order to detect differentially abundant taxa across groups (Segata et al., 2011). Microbial functions were predicted using Phylogenetic Investigation of Communities by Reconstruction of Unobserved States (PICRUSt), based on high-quality sequences (Langille et al., 2013). OTUs were normalized by copy number, and metagenomic prediction was performed based on Kyoto Encyclopedia of Genes and Genomes (KEGG) (Kanehisa et al., 2012).

### Statistical Analysis

Data are shown as mean and SEM. Differences in average body weight, average daily gain (ADG), FCR, alpha diversity indices, and the relative abundance of bacterial taxa among treatment groups were analyzed using one-way ANOVAs, followed by Duncan multiple comparison tests, in SPSS v18.0 (Chicago, IL, United States). The relative abundances of the 10 most abundant phyla, the relative abundances of genera and species, and bacterial functional pathways were analyzed using LEfSe^1^ (Segata et al., 2011). The alpha value of the Kruskal–Wallis test among classes was set to 0.5, and the log10 linear discriminant analysis (LDA) score was limited to 2.0.

### Data Access

All raw sequences were deposited in the NCBI Sequence Read Archive (accession number SRP188289).

## Results

### Growth Performance

PE or VIRG dietary supplementation affected broiler growth performance (Table 2). On day 28, chicken body weight in the HPE group was higher (1264.3 g) than that in the CT group (1076.1 g) (*P* < 0.05). On d42, the final body weight of broilers treated with HPE (2536.57 g) and VIRG (2520.77 g) was increased 4.66 and 4.03% respectively as compared to that of the CT group (2423.0 g). However, the difference was insignificant (*P* = 0.20). Treatment with PEs or VIRG significantly decreased the feed-to-gain ratio over days 15–28 and 29–42 (*P* < 0.01). However, there were no significant differences in body weight and the feed-to-gain ratio over days 1–14 (*P* > 0.05).

**Table 2:** Effects of plant extracts and virginiamycin on broiler growth performance^1^.

	CT	VIRG	LPE	HPE	SEM	*P*-value
**Average body weight**						
at 14 days (g/bird)	306.26	298.22	308.18	312.60	4.0	0.68
at 28 days (g/bird)	1076.1^b^	1120^b^	1172.7^ab^	1264.3^a^	22.0	0.01
at 42 days (g/bird)	2423	2520.7	2504.3	2536.6	20.2	0.20
**Average daily gain (g/d)**						
Days 1–14	19	18	19	19	0.288	0.51
Days 15–28	55^c^	58^c^	60^ab^	68^a^	1.636	0.03
Days 29–42	96^ab^	100^a^	97^ab^	91^b^	1.495	0.19
**Average feed intake (g/d)**						
Days 1–14	27	24	27	30	0.014	0.72
Days 15–28	92^a^	84^b^	86^b^	91^c^	0.031	0.02
Days 29–42	156^a^	134^b^	141^b^	150^a^	0.044	0.04
**Feed to gain ratio**						
Days 1–14	1.46	1.38	1.30	1.36	0.060	0.68
Days 15–28	1.74^a^	1.44^c^	1.56^bc^	1.60^b^	0.050	0.001
Days 29–42	1.86^a^	1.65^b^	1.64^b^	1.64^b^	0.081	0.001

*^*1*^Each value represents the mean of six replicates. CT, basal diet (control); VIRG, basal diet supplemented with 30 mg/kg virginiamycin; LPE, basal diet supplemented with 200 mg/kg plant extracts; HPE, basal diet supplemented with 400 mg/kg plant extracts. ^*a,b*^Different letters in the same row indicate significant differences between the respective means (*P* < 0.05)*

### Diversity and Structure of Intestinal Microbiota

**FIGURE 1:**
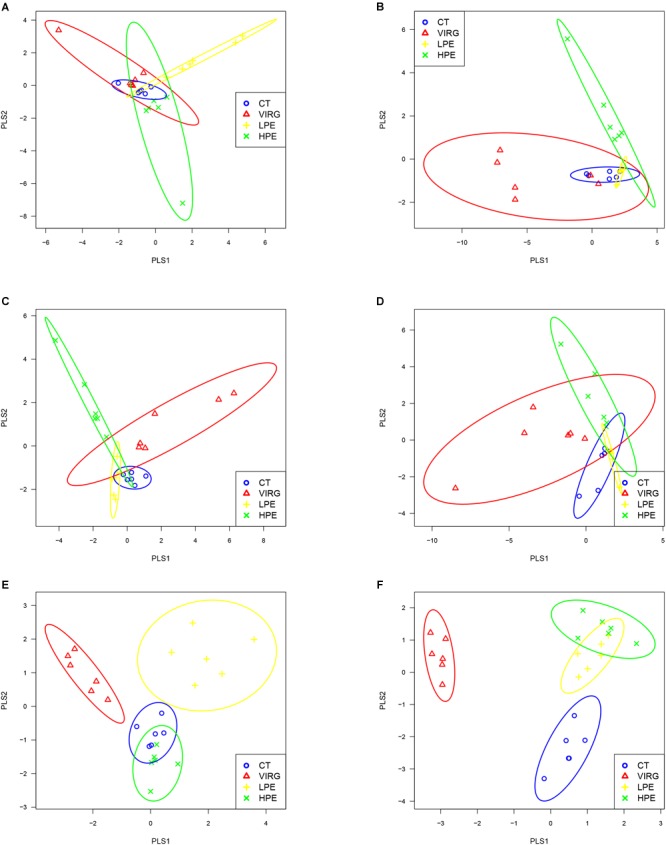
Principal coordinate analysis (PLS-DA), based on the weighted unifrac distances of 16S rRNA bacterial sequences from the intestines of broilers. **(A,B)** PLS-DA of duodenal bacteria at **(A)** day 14 or **(B)** day 28. **(C,D)** PLS-DA of ileal bacteria at **(C)** day 14 or **(D)** day 28. **(E,F)** PLS-DA of cecal bacteria at **(E)** at day 14 or **(F)** day 28. CT, basal diet (control); VIRG, basal diet supplemented with 30 mg/kg virginiamycin; LPE, basal diet supplemented with 200 mg/kg plant extracts; HPE, basal diet supplemented with 400 mg/kg plant extracts.

Raw reads were denoised and then cleaned to remove chimeras and low-quality sequences. A total of 5,254,194 quality-controlled reads were obtained, an average of 36,487 sequences per sample. An average of 1080 OTUs per sample (based on 97% sequence similarity) was identified, with an average sequence length of 494.5 bp. The bacterial OTU numbers, microbial richness, and diversity are presented in Table 3. In the HPE group, the Shannon and Simpson diversity indices in the ileum were significantly lower than those of the CT group on day 14 only (*P* < 0.05; Table 3). However, in the VIRG group, the Shannon and Simpson diversity indices in the duodenum were significantly greater than those of the CT group on days 14 and 28 (*P* < 0.05; Table 3). No significant differences in richness and diversity were observed in the ceca among all of the treatments groups on days 14 and 28 (*P* > 0.05). Principal coordinate analysis indicated that microbes from the ceca of the HPE and CT groups formed distinct clusters, while microbes from both the ileum and ceca of the VIRG and CT groups formed distinct clusters (Figure 1). No differences in duodenal microbial communities among treatment groups were identified on days 14 and 28 (Figure 1A,B).

**Table 3:** Effects of plant extracts and virginiamycin on the diversity of the intestinal microbiota^1^.

			CT	VIRG	LPE	HPE	SEM	*P*-value
Day 14	Duodenum	OTU	957.17	914.50	801.33	849.	23.47	0.08
		Chao1	1121	1017	918	968.4	29.22	0.07
		ACE	1201	1067	976.9	1040	32.50	0.09
		Simpson	0.87^ab^	0.96^a^	0.86a^b^	0.79^b^	0.02	0.03
		Shannon	6.13^b^	7.10^a^	5.81^b^	5.38^b^	0.20	0.01
	Ileum	OTU	1176	1099	1037.17	930.5	35.57	0.08
		Chao1	1330	1223	1156.37	1025	42.40	0.07
		ACE	1399	1286	1236.65	1093	43.83	0.09
		Simpson	0.93^a^	0.93^a^	0.90^a^	0.82^b^	0.02	0.02
		Shannon	6.99^a^	7.06^a^	6.49^ab^	5.61^b^	0.19	0.01
	Cecum	OTU	1366	1101	1278	1397	56.67	0.26
		Chao1	1516	1185	1455	1574	66.77	0.17
		ACE	1533	1198	1517	1607	68.87	0.15
		Simpson	0.96	0.98	0.95	0.96	0.01	0.55
		Shannon	7.90	7.98	7.58	7.92	0.19	0.89
Day 28	Duodenum	OTU	647.8	836	660	835	49.85	0.36
		Chao1	745.9	951	760	967	58.18	0.39
		ACE	809.5	1004	818	1044	62.68	0.44
		Simpson	0.47^b^	0.82^a^	0.57^b^	0.65^ab^	0.05	0.03
		Shannon	2.99^b^	5.84^a^	3.64^b^	4.41^ab^	0.38	0.04
	Ileum	OTU	945.2	736	1062	901	43.81	0.06
		Chao1	1066	819	1175	1020	48.02	0.05
		ACE	1144^a^	858^b^	1240^a^	1094^ab^	51.36	0.04
		Simpson	0.76	0.86	0.83	0.69	0.03	0.20
		Shannon	5.25	5.81	6.04	4.68	0.27	0.31
	Cecum	OTU	1256	1215	1070	1147	38.96	0.37
		Chao1	1466	1374	1173	1223	49.44	0.13
		ACE	1498	1425	1190	1229	52.32	0.10
		Simpson	0.95	0.97	0.96	0.98	0.01	0.28
		Shannon	7.47	7.51	7.26	7.82	0.15	0.62

*^*1*^Each value represents the mean of six replicates. CT, basal diet (control); VIRG, basal diet supplemented with 30 mg/kg virginiamycin; LPE, basal diet supplemented with 200 mg/kg plant extracts; HPE, basal diet supplemented with 400 mg/kg plant extracts. ^*a,b*^Different letters in the same row indicate significant differences between the respective means (*P* < 0.05).*

### Relative Abundance of Bacterial Taxa in the Intestinal Microbiota

**FIGURE 2:**
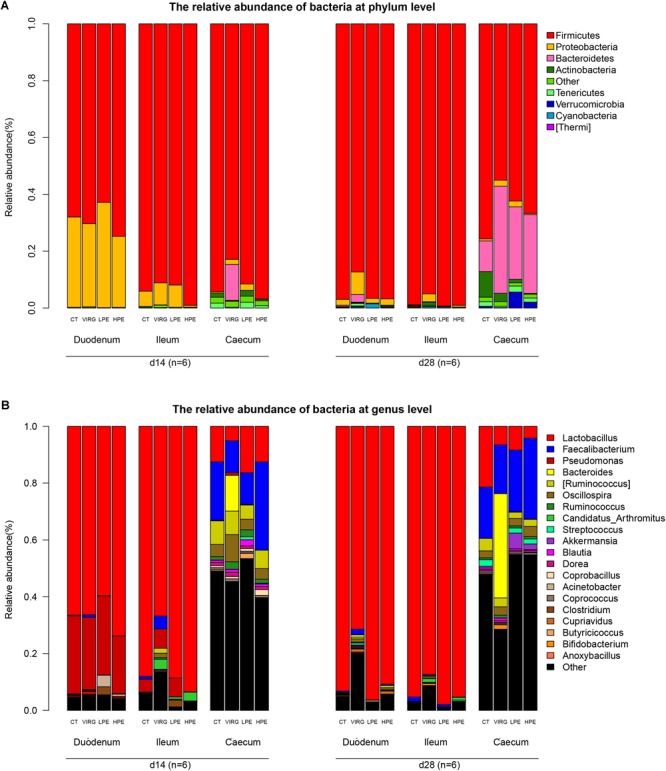
Effects of plant extracts and virginiamycin on duodenal, ileal, and cecal microbiota composition at phylum and genus level. Relative abundances of bacterial phyla **(A)** and genera **(B)** in four treatment groups. CT, basal diet (control); VIRG, basal diet supplemented with 30 mg/kg virginiamycin; LPE, basal diet supplemented with 200 mg/kg plant extracts; HPE, basal diet supplemented with 400 mg/kg plant extracts. Intestinal chyme was collected from six birds per group for this analysis (*n* = 6).

Firmicutes was the dominant bacterial phylum in duodenum and ileum (75–95% of all OTUs; Figure 2A). In the cecum, the microbiota included 55–70% Firmicutes, 25–30% Bacteroidetes, and 3% other phyla. Dietary treatments affected relative bacterial abundance at the phylum level (Figure 2A). Compared with the CT group, Firmicutes was more abundant in the intestinal microbiota of the HPE group on day 14. On day 28, Bacteroidetes was more abundant in the cecal microbiota of the HPE group as compared to the CT group, and Actinobacteria was less abundant. However, Firmicutes was less abundant in the intestinal microbiota of the VIRG treatment group as compared to the control group (except in the duodenum on day 14). In addition, VIRG treatment increased the relative proportion of Proteobacteria in the ileum and Bacteroidetes in the cecum.

*Lactobacillus* was the dominant genus in the duodenal and ileal microbial communities (Figure 2B). On day 14, the duodenal microbiotas of the broilers were 50–70% *Lactobacillus* and 20–27% *Pseudomonas*. On day 28, almost 90% of all bacteria in the duodenum and ileum samples were *Lactobacillus.* The cecal microbiota included 10–20% *Lactobacillus* and 10–20% *Faecalibacterium*. The SPSS analysis of all bacterial genera with relative abundances > 0.1% is shown in Table 4. The results indicated that 28 days of dietary supplementation with PEs significantly decreased the relative abundance of *Lactobacillus* (from 21.48% in the CT group to 8.41 and 4.2% in the LPE and HPE groups, respectively; *P* < 0.05), and significantly increased the relative abundances of *Faecalibacterium* (from 18–20% in the CT group to 28–31% in the HPE group; *P* < 0.01) and Rikenellaceae (from 10.83% in the CT group to 25–27% in the LPE and HPE groups; *P* < 0.01) in the cecal microbiota. Dietary supplementation with VIRG for 14 and 28 days also significantly decreased the relative abundance of *Lactobacillus* in the broiler ileum and cecum samples as compared to the CT group, and increased the relative abundance of *Bacteroides* in the cecum samples (*P* < 0.01). Supplementation with either VIRG or PEs also increased the relative abundance of Lachnospiraceae in the cecum of broilers on day 14.

**Table 4:** Effects of plant extracts and virginiamycin on the relative abundance of bacterial genera (%)^1^.

Genus	Treatment	Day 14			Day 28		
		Duodenum	Ileum	Cecum	Duodenum	Ileum	Cecum
*Lactobacillus*	CT	66.48	88	12.7	93.15	95.25	21.48
	VIRG	66.35	66.55	5.13	71.21	87.3	6.58
	LPE	59.63	88.6	16.45	96.33	97.9	8.41
	HPE	73.8	93.5	12.61	90.73	95.3	4.2
	*P*-value	0.633	0.043	0.439	0.033	0.215	0.029
*Faecalibacterium*	CT	0.21	0.983	20.53	0.4	1.51	18
	VIRG	0.9	4.63	11.25	1.98	0.4	17.13
	LPE	0.033	ND^2^	11.25	0.16	0.86	21.71
	HPE	0.033	ND	31	0.41	0.06	28.46
	*P*-value	0.461	0.457	0.117	0.057	0.607	0.413
*Unclassified Ruminococcaceae*	CT	0.3	2.98	24.21	1	0.43	10.38
	VIRG	0.733	4.63	17.21	3.73	1.53	9.81
	LPE	0.033	ND	21.05	0.1	0.166	9.38
	HPE	0.116	ND	15.88	1.83	0.05	10.6
	*P*-value	0.514	0.229	0.648	0.258	0.455	0.98
*Pseudomonas*	CT	27.51	4.58	0.183	ND	ND	ND
	VIRG	25.36	6.8	0.933	ND	0.022	ND
	LPE	27.91	ND	0.2	ND	ND	ND
	HPE	20.26	0.067	0	ND	ND	ND
	*P*-value	0.635	0.104	0.473		0.013	
Unclassified Rikenellaceae	CT	0.016	ND	ND	ND	ND	10.83
	VIRG	ND	ND	ND	ND	ND	0.88
	LPE	0.016	ND	ND	ND	ND	25.43
	HPE	ND	ND	ND	ND	ND	27.63
	*P*-value	0.582					0.0001
*Bacteroides*	CT	ND	ND	ND	ND	ND	ND
	VIRG	ND	ND	12.53	0.783	0.067	36.7
	LPE	ND	ND	ND	ND	ND	0.116
	HPE	ND	ND	ND	ND	ND	0.083
	*P*-value	ND	ND	0.118	ND	0.022	0.0001
Unclassified Lachnospiraceae	CT	0.083	0.25	3.93	0.3	ND	2.73
	VIRG	0.066	2.23	8.3	1.48	ND	4.86
	LPE	ND	ND	5.51	0.016	ND	3.2
	HPE	ND	ND	6.06	0.76	ND	2.5
	*P*-value	0.396	0.212	0.045			0.027

*^*1*^Each value represents the mean of six replicates. CT, basal diet (control); VIRG, basal diet supplemented with 30 mg/kg virginiamycin; LPE, basal diet supplemented with 200 mg/kg plant extracts; HPE, basal diet supplemented with 400 mg/kg plant extracts. ^*2*^ND indicates a relative abundance < 0.01%.*

**FIGURE 3:**
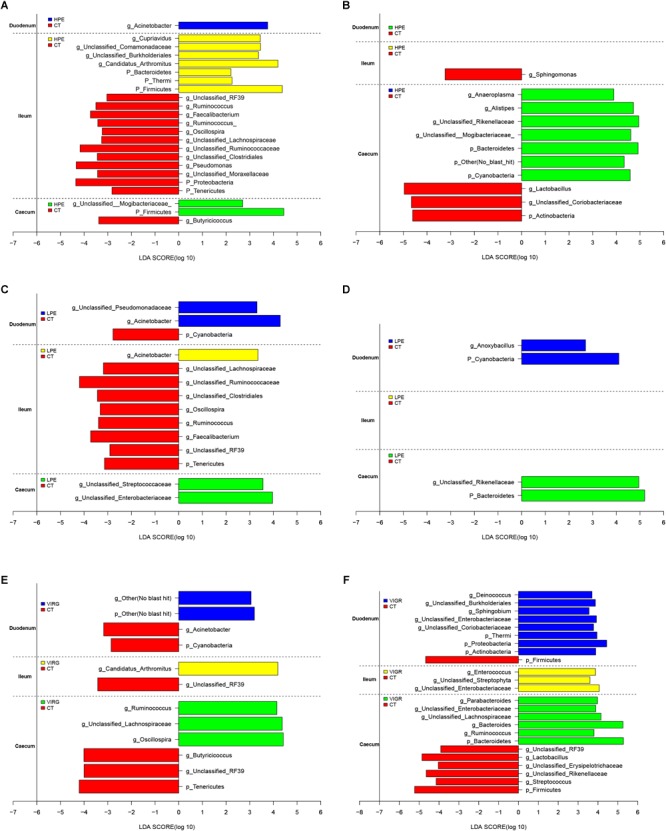
Histograms showing the results of the linear discriminant analysis effect size (LEfSe) measurements, identifying the most differentially abundant phyla and genera between the PE/VIRG and CT groups. **(A,B)** Representation of the LEfSe results, showing different phyla and genera in the duodenum, ileum, and cecum samples from the HPE and CT chickens at day 14 and 28. **(C,D)** Representation of the LEfSe results, showing different phyla and genera in the duodenum, ileum, and cecum samples from the LPE and CT chickens at day 14 and 28. **(E,F)** Representation of the LEfSe results, showing different phyla and genera in the duodenum, ileum, and cecum samples from the VIRG and CT chickens at day 14 and 28. The CT chickens are indicated with a negative LDA scores (LDA > 2; *P* < 0.05). CT, basal diet (control); VIRG, basal diet supplemented with 30 mg/kg virginiamycin; LPE, basal diet supplemented with 200 mg/kg plant extracts; HPE, basal diet supplemented with 400 mg/kg plant extracts.

The LEfSe results suggested that supplementation with PEs modified the microbial composition of ileum on day 14 and that of cecum on day 28 (Figure 3; LDA > 2; *P* < 0.05). On day 14, the relative abundances of three phyla (Firmicutes, Bacteroidetes and Thermi) and four genera (e.g., *Candidatus Arthromitus* and unclassified Burkholderiales) increased in the ileal microbiotas of the HPE group as compared to the CT group, and the relative abundances of two phyla (Proteobacteria and Tenericutes) and 10 genera (e.g., *Ruminococcus, unclassified RF39*, Faecalibacterium, and *Oscillospira*) decreased. On day 28, the relative abundances of two phyla (Bacteroidetes and Cyanobacteria) and three genera (*Alistipes*, unclassified Rikenellaceae, unclassified Mogibacteriaceae, and *Anaeroplasma*) increased in the cecal microbiota of the HPE group as compared to the CT group, and the relative abundances of one phylum (Actinobacteria) and two genera (*Lactobacillus* and unclassified Coriobacteriaceae) decreased (Figure 3A,B). In addition, the relative abundances of Bacteroidetes and several genera of unclassified *Rikenellaceae* increased in the cecal microbiotas of the LPE group on day 28 as compared to the CT group (Figure 3D). On day 14, the relative abundances of the genera *Ruminococcus, Lachnospiraceae*, and *Oscillospira* were higher in the cecal microbiota of the VIRG group as compared to the CT group (Figure 3E). On day 28, the relative abundances of one phylum (Firmicutes) and four genera (*Lactobacillus*, unclassified RF39, unclassified Rikenellaceae, and unclassified Erysipelotrichaceae) were lower in the duodenal and cecal microbiotas of the VIRG group as compared to the CT group, and the relative abundances of one phylum (Bacteroidetes) and four genera (*Bacteroides*, Enterobacteriaceae, *Ruminococcus*, and Lachnospiraceae) were higher in the cecal microbiotas of the VIRG group as compared to the CT group (Figure 3F).

### Changes in Intestinal Microbiota Function

**FIGURE 4:**
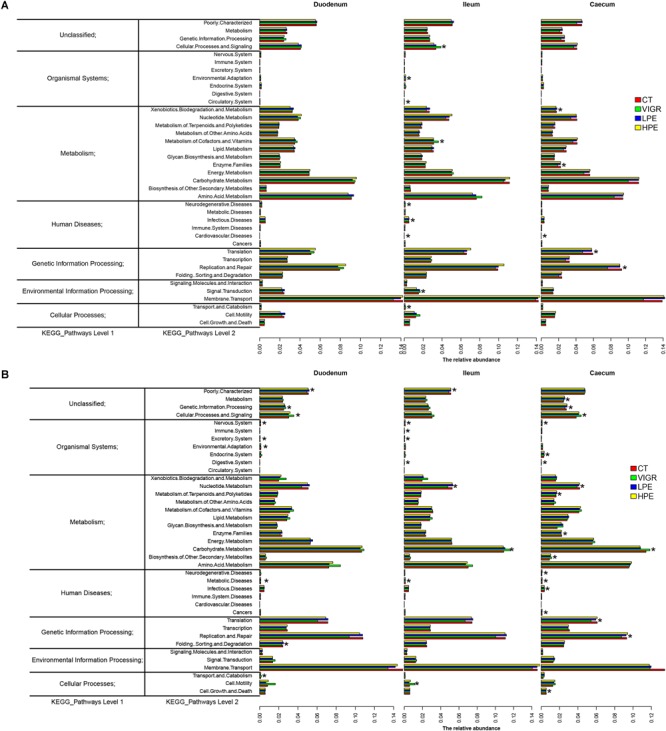
Genes overrepresented in the level 2 KEGG pathways in the duodenal, ileal, and cecal microbiotas of broilers on **(A)** day 14 and **(B)** day 28. CT, basal diet (control); VIRG, basal diet supplemented with 30 mg/kg virginiamycin; LPE, basal diet supplemented with 200 mg/kg plant extracts; HPE, basal diet supplemented with 400 mg/kg plant extracts.

The predicted functions of the intestinal microbiota were identified using PICRUSt. At KEGG level 2 (Figure 4), several functions of the ileal microbiota were differentially enriched among treatments after 14 days, including cellular processes and signaling, metabolism of cofactors and vitamins, and infectious diseases. Several functions of the cecal microbiota were also differentially enriched on day 14, including the xenobiotics biodegradation metabolism and enzyme families (Figure 4A). Several functions associated with the digestive system and metabolic disease were differentially enriched in the intestinal microbiota among treatments after 28 days. In the VIRG group at days 14 and 28, the ileal and cecal microbiotas were enriched in functions associated with the carbohydrate metabolism and the digestive system (Figure 4B).

**FIGURE 5:**
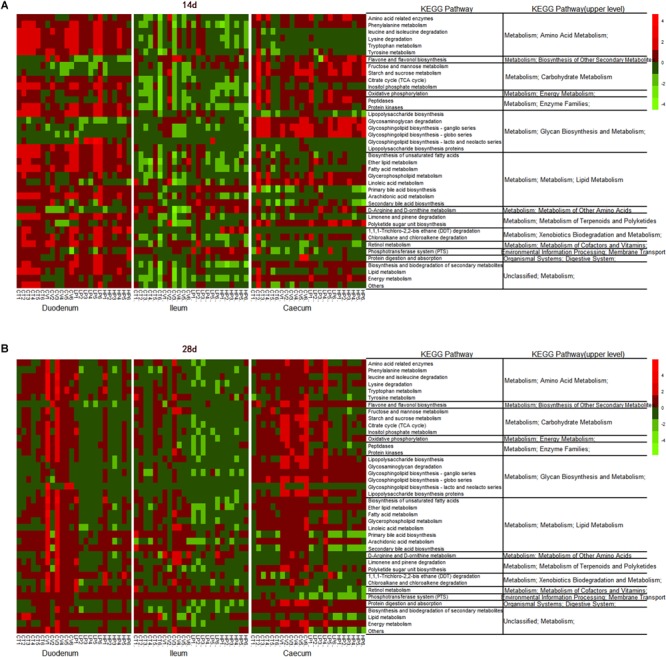
Heatmap of relative abundance of predicted functional KEGG pathways of level 3 of the duodenal, ileal, and cecal microbiota of broilers on **(A)** day 14, and **(B)** day 28 by the LEfSe test (LDA > 2; *P* < 0.05). CT, basal diet (control); VIRG, basal diet supplemented with 30 mg/kg virginiamycin; LPE, basal diet supplemented with 200 mg/kg plant extracts; HPE, basal diet supplemented with 400 mg/kg plant extracts.

The LEfSe analysis of KEGG level 3 identified 145 KEGG categories related to metabolic pathways; of these 40 were significantly differentially enriched among treatment groups (LDA > 2; *P* < 0.05). The heat map of metabolic pathway enrichment across treatment groups showed that the amino acid and lipid metabolisms in the duodenum samples were significantly affected in the HPE and VIRG groups (Figure 5). In the HPE group at day 14, the cecal microbiota was more enriched in functions involved in amino-acid-related enzymes, the D-Arginine and D-ornithine metabolism and carbohydrate metabolism (i.e., fructose and mannose metabolism, starch and sucrose metabolism, and the citrate cycle), as compared to the CT group (Figure 5A). In the VIRG group at day 28, the duodenal and cecal microbiotas were more enriched in functions involved in the amino acid, lipid, and carbohydrate metabolisms than those of the CT group (Figure 5B).

**FIGURE 6:**
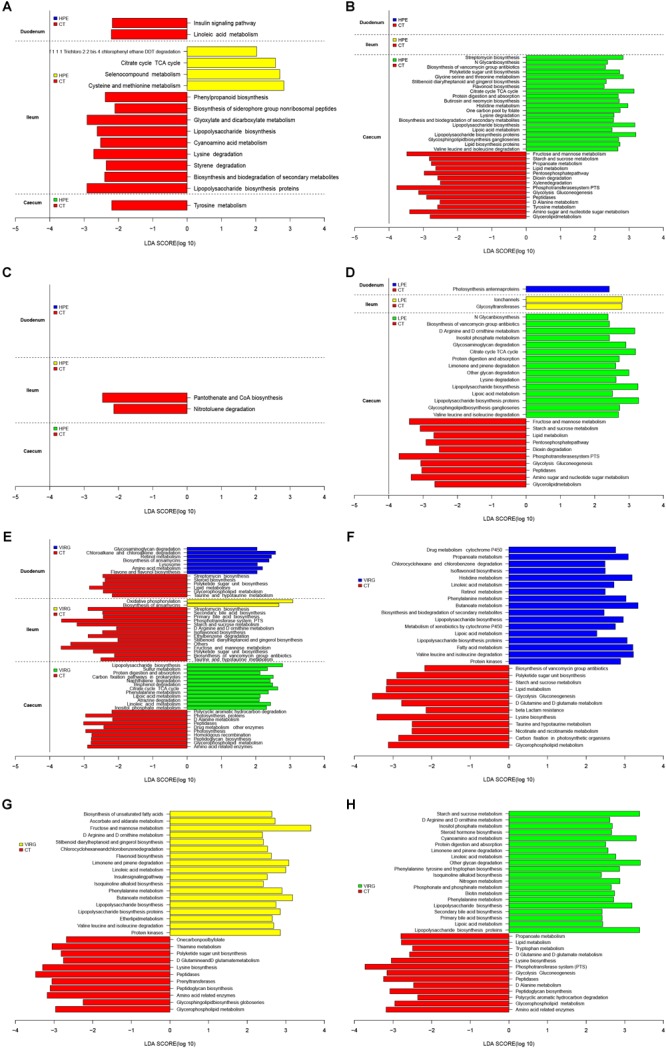
PICRUSt analysis of differences in metabolic functions among treatments, based on LEfSe results (LDA > 2; *P* < 0.05). **(A,B)** Differences in the metabolic functions of the intestinal microbiota between HPE and CT group chickens at **(A)** day 14 and **(B)** day 28. **(C,D)** Differences in the metabolic functions of the intestinal microbiota between LPE and CT group chickens at **(C)** day 14 and **(D)** day 28. **(E)** Differences in the metabolic functions of the intestinal microbiota between VIRG and CT group chickens at day 14. **(F–H)** Differences in the metabolic functions of the **(F)** duodenal, **(G)** ileal, and **(H)** cecal microbiota between VIRG and CT group chickens at day 28. CT, basal diet (control); VIRG, basal diet supplemented with 30 mg/kg virginiamycin; LPE, basal diet supplemented with 200 mg/kg plant extracts; HPE, basal diet supplemented with 400 mg/kg plant extracts.

LEfSe was used to analyze differences in the enrichment of 145 identified level-3 metabolic pathways between the CT and HPE/VIRG groups (Figure 6). At day 14 in the ileal microbiota of the HPE group, functions associated with the cysteine and methionine metabolism, the citrate cycle (TCA cycle), and the selenocompound metabolism were enriched compared to the CT group (Figure 6A). At day 28, 20 metabolic pathways were more enriched in the HPE cecal microbiota as compared to the CT cecal microbiota (Figure 6B), including protein digestion/absorption, various metabolisms (including amino acid, lipid, carbohydrate, histidine, glycine, serine and threonine, lysine, valine, and lipoic acid), leucine and isoleucine degradation, lipid biosynthesis proteins, and the citrate cycle (TCA cycle). In the LPE group at 28 days, 15 metabolic pathways were enriched in the cecal microbiota (Figure 6C,D). Interestingly, pathways associated with protein digestion and absorption, amino acid metabolism, lipopolysaccharide biosynthesis proteins, the citrate cycle (TCA cycle), and lipoic acid metabolism were upregulated in both the LPE and HPE groups.

In the intestinal microbiota of the VIRG group at day 14, 21 identified metabolic pathways were enriched as compared to the CT group (7 in the duodenum, 2 in the ileum, and 12 in the cecum; Figure 6E). In addition, 55 categories (17 in the duodenum, 18 in the ileum, and 20 in the cecum) were significantly enriched in the VIRG group on day 28 (Figure 6F). Thus, dietary supplementation with PEs and VIRG affected important predicted functions of the intestinal microbiota.

## Discussion

The present study showed that treatment with PEs increased the ADGs of broiler chickens during the growth phase, and improved conversion feed rates during all of the phases. Several previous studies have shown that PEs have positive effects on body weight gain and the feed-to-gain ratio in chickens (Jamroz and Kamel, 2002; Ciftici et al., 2005; Jang et al., 2007; Zhang et al., 2009: Khattak et al., 2014). However, some authors have found that PE treatment only improved chicken body weight gain, and did not affect the feed-to-gain ratio (Ocak et al., 2008; Bozkurt et al., 2009). Finally, Jamroz et al. (2005) and Amad et al. (2011) showed that PEs improved the feed conversion rate, but had no effect on body weight or feed intake.

Although it remains unclear precisely how PEs supplementation improves chicken growth, several mechanisms have been proposed, including modifications of intestinal microbial ecology (e.g., reducing pathogenic stress or increasing the abundance of beneficial microorganism in the gut), increasing digestive enzyme secretions, and improving nutrient absorption (Windisch et al., 2008; Awaad et al., 2014; Wlodarska et al., 2015; Zeng et al., 2015; Stevanović et al., 2018). *In vitro* experiments have shown that PEs have antibacterial, antifungal, and antiviral properties; PEs are more active against gram-positive bacteria than against gram-negative bacteria (Burt, 2004; Windisch et al., 2008; Zeng et al., 2015). Some recent *in vivo* experiments have suggested that PEs modify the composition of the intestinal microbiota, increasing the relative abundance of Firmicutes in the gut (Salaheen et al., 2017; Li et al., 2018), as well as the abundance of *Clostridiales*, Ruminococcaceae, and Lachnospiraceae in the PE-treatment group (Diaz Carrasco et al., 2018). Here, the Simpson and Shannon diversity indices in the ileum microbiota decreased in the HPE group. This was consistent with the results of Yin et al. (2017), which showed that supplementation with essential oils decreased the Shannon and Simpson diversity indices. Moreover, HPE treatment increased the relative abundances of Firmicutes in duodenal and ileal microbiotas, and increased the relative abundances of Bacteroidetes and several genera (unclassified Rikenellaceae, *Faecalibacterium* and *Lachnospiraceae*) in the cecal microbiota.

Recent studies have identified correlations between intestinal microbial composition and the efficiency of energy extraction in humans and animals (Turnbaugh et al., 2006; Ley et al., 2007). Firmicutes and Bacteroidetes are two of the dominant bacterial phyla in chicken. The ratio of Firmicutes abundance to Bacteroidetes abundance in the gut microbiota has been linked to the efficiency of energy harvesting in humans and various animals (Turnbaugh et al., 2006; Ley et al., 2007; Zhao et al., 2015). Firmicutes species are associated with the decomposition of polysaccharides and the production of butyrate. Bacteroidetes species degrade complex carbohydrates and synthesize propionate via the succinate pathway (Turnbaugh et al., 2006; Louis et al., 2014). *Alistipes* and unclassified Rikenellaceae fall into Bacteroidales. *Alistipes*, belong to the main member within family Rikenellaceae, are generally considered beneficial to the host gut (Abe et al., 2012). These bile-resistant bacteria have saccharolytic and proteolytic properties, produce acetic acid by producing fibrinolysin, digest gelatin, and ferment carbohydrates (Rautio et al., 2003). The genus *Faecalibacterium* includes gram-positive, anaerobic bacteria, which produce butyrate and other short-chain fatty acids through the fermentation of dietary fiber in the host gut (Martín et al., 2017). The *Lachnospiraceae* (order Clostridiales), degrade plant materials and produce bacteriocins, butyric acid, and lactate (Turnbaugh et al., 2006; Koh et al., 2016). In addition, *Lachnospiraceae* and *Faecalibacterium* were associated with improved FCRs and BWG rates in birds (Stanley et al., 2016). Thus, several studies investigating the properties and functions of these bacterial genera have suggested that these microorganisms might be useful as poultry probiotics (Torok et al., 2011; Stanley et al., 2016; Martín et al., 2017).

Similar to the VIRG group, the relative abundances of *Lactobacillus* in the cecal microbiotas of LPE and HPE groups decreased. These results were consistent with those of Thapa et al. (2012) and Placha et al. (2014), who reported that some beneficial commensal bacteria (such as *Lactobacillus*) were less abundant after PE treatment. In contrast, other studies have shown that PE treatment increased the relative taxon abundance of *Lactobacillales* (Viveros et al., 2011; Du et al., 2016; Yin et al., 2017; Li et al., 2018). Here, we found that *Lactobacillus* abundance decreased in the intestine after VIRG treatment. This was consistent with previous studies, which showed that the relative abundance of *Lactobacillus* decreased in the intestines of chickens after administration of antibiotics (Lan et al., 2005; Guban et al., 2006; Danzeisen et al., 2011; Allen and Stanton, 2014; Mancabelli et al., 2016; Yu et al., 2018). *Lactobacillus*, which is used as a probiotic, has a beneficial effect on host intestinal health and growth (Huang et al., 2004). However, some Lactobacilli were associated with poor growth performance and FCR (Torok et al., 2011; Stanley et al., 2012). Indeed, some strains of *Lactobacillus* are sold as weight loss probiotics and others are reported to have the ability to reduce obesity by decreasing appetite and food consumption (Fåk and Bäckhed, 2012). Moreover, De Boever et al. (2000) found that *Lactobacillus reuteri* decreased lipid absorption and consequently, caused dietary energy losses, possibly by stimulating bacterial bile salt hydrolysis.

The observed shifts in the diversity and composition of the chicken intestinal microbiota may have affected gut microbial metabolism, gut physiological function, host health, and growth. The PICRUSt results showed that, as compared to the CT group, the PE-treated ileal microbiota was enriched in functions associated with cofactors and vitamin metabolism as well as infectious diseases. In addition, PE treatment enriched 15 pathways associated with metabolic function in the cecal microbiota of the LPE group, and 20 pathways associated with metabolic function in the cecal microbiota of the HPE group. These pathways included protein digestion and absorption, amino acid metabolism, lipid biosynthesis proteins, lipopolysaccharide biosynthesis (proteins), citrate cycle (TCA cycle), and lipoic acid metabolism. These results were consistent with those of Li et al. (2018), who reported that essential oils increased protein biosynthesis, as well as lipid and amino acid metabolism, in the colon microbiotas of weaned piglets. Similar alterations in intestinal microbial function may have caused the performance improvements observed in the chickens fed PEs. Several previous studies have also shown that antibiotic treatment affected the predicted functions enriched in the intestinal microbiota (Choi et al., 2017). Danzeisen et al. (2011) demonstrated that *Roseburia* and *Lactobacillus* reduced and transport system genes were enriched in the cecal microbiotas of broilers fed VIRG supplements. The current study demonstrated that, similar to VIRG, PEs enhanced broiler growth performance, possibly due to improved intestinal microbial composition and metabolic function.

## Conclusion

The results of the present study showed that dietary supplementation with PEs or virginiamycin altered the diversity, composition, and function of the intestinal microbiota. PE treatment enhanced the relative abundances of Firmicutes in duodenal and ileal microbiotas, and the relative abundances of unclassified Rikenellaceae, *Faecalibacterium*, and *Lachnospiraceae* in the cecal microbiota. The enhancement of several metabolic pathways in the intestinal microbiota, such as protein digestion/absorption, and amino acid metabolism, might have resulted in the improved chicken growth performance. These results provide a theoretical framework for the use of PEs as an alternative to antibiotics in broiler production.

## Author Contributions

NZ and BL designed the experiments and wrote the manuscript. JW, KC, and QZ performed the experiments and collected the samples. JW and LY analyzed the data. NZ and BL were responsible for the final content. All of the authors have read and approved the final manuscript.

## Conflict of Interest Statement

QZ was employed by Guangdong Ruisheng Technology Co., Ltd. The remaining authors declare that the research was conducted in the absence of any commercial or financial relationships that could be construed as a potential conflict of interest.

## Footnotes

1 http://huttenhower.sph.harvard.edu/galaxy
